# Efficient catalytic degradation of methylene blue by a novel Fe^3+^-TiO_2_@CGS three-dimensional photoelectric system

**DOI:** 10.3389/fchem.2022.1065003

**Published:** 2022-12-06

**Authors:** Jian Li, Yufei Wang, Fanhui Guo, Juan Chen, Jinxi Wang, Xiaoyong Fan, Baoning Li, Santosh Kumar Verma, Qingbo Wei, Long Yan, Jianjun Wu

**Affiliations:** ^1^ School of Chemical Engineering and Technology, National Engineering Research Center of Coal Preparation and Purification, China University of Mining and Technology, Xuzhou, China; ^2^ School of Chemistry and Chemical Engineering, Yulin University, Yulin, China; ^3^ School of Chemistry and Chemical Engineering, Yan’an University, Yan’an, China

**Keywords:** three-dimensional Fe^3+^TiO_2_@CGS, photoelectric system, catalytic degradation, methylene blue, waster water treament

## Abstract

In this study, a novel three-dimensional photoelectric system was designed and constructed for the degradation of methylene blue (MB) *via* photocatalysis, electrocatalysis, and photoelectric catalysis. To this end, a Ti/RuO_2_-IrO_2_-SnO_2_-CeO_2_ electrode was prepared *via* a thermal oxidation coating method and used as a dimensionally-stable anode (DSA). The cathode was made of a titanium sheet with Fe^3+^-doped TiO_2_ loaded on coal gasification slag (CGS) (Fe^3+^-TiO_2_@CGS) as a photocatalyst. The factors affecting the degradation efficiency, such as the supporting electrolyte, current density, and initial pH were systematically investigated. The results revealed Fe^3+^-TiO_2_@CGS three-dimensional photoelectric system exhibiting efficient synergistic performance of photocatalysis and electrocatalysis with a synergistic factor of 1.11. Photo-generated holes (h^+^) were generated by light irradiation and direct anodic oxidation. Furthermore, hydroxyl radicals (HO·) radicals were induced *via* other pathways. Such active species showed highly-oxidizing abilities, beneficial to the degradation of methylene blue (MB). The representative Fe^3+^-TiO_2_@CGS three-dimensional photoelectric system showed super high degradation efficiency at pH 11 and current density of 18.76 mA cm^−2^. Using NaCl as a supporting electrolyte, the degradation yield reached 99.98% after 60 min of photoelectrical treatment. Overall, the novel Fe^3+^-TiO_2_@CGS three-dimensional photoelectrical system looks very promising for the highly efficient catalytic degradation of organic contaminants.

## Graphical Abstract

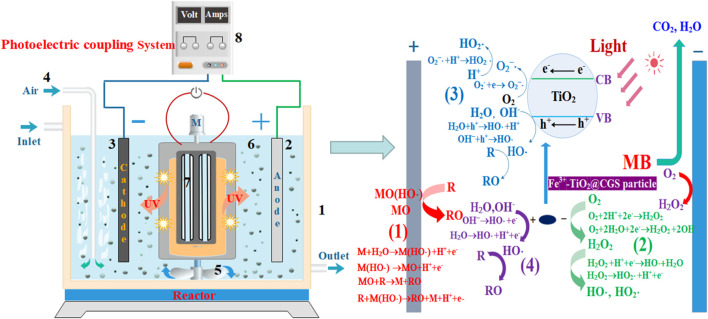

## 1 Introduction

In recent years, with the continuous advancement of China’s social development process, China’s annual sewage discharge has continued to increase. In the past 5 years, the annual sewage discharge has been about 400 billion cubic meters, accounting for about 1/8 of the global sewage discharge (Meng et al., 2021; Sun et al., 2022). Highly toxic organic contaminants in industrial wastewater pose severe threats to the sustainable development of natural water resources. Therefore, water protection has become the focus of human society (Li and Qian, 2018; Sun et al., 2019; Shi et al., 2020; Wang et al., 2020). At present, the industrial wastewater treatment methods mainly includes physical, chemical, biological and membrane separation method according to the properties of the wastewater. Among them, the chemical method of advanced oxidation technology is widely used in this field, which including wet oxidation, supercritical oxidation, ultrasonic degradation, electrochemical, and photocatalytic oxidation method, et al. However, it is difficult to directly achieve the requirements in the treatment of industrial wastewater through a single method (Dutta et al., 2021; Dębowski et al., 2022). Biological treatment has commonly been used as a technology for cleaning toxic organic wastewater. However, this technology is relatively inefficient for highly toxic contaminants requiring severe degradation conditions (Machineni, 2020; Miguel et al., 2020). To address these problems, advanced oxidation methods have been investigated since these can directly or indirectly mineralize the organic or macromolecular contaminants that are extremely toxic and difficult to dissolve (Glaze et al., 1987; Ayoub et al., 2010; Zeng et al., 2012; Oturan and Aaron, 2014; Ge et al., 2016; Savun-Hekmolu, 2021).

The electrochemical oxidation technology is an advanced oxidation method advantageous in terms of effective treatment and simple operation, thereby being considered one of the most promising technologies for water treatment. The electrochemical oxidation technology has been applied for the treatment of dye wastewater (Liu et al., 2011; Nidheesh and Gandhimathi, 2012; Kruthika et al., 2013; Payra et al., 2019; Sathishkumar et al., 2019), coking wastewater (Wang et al., 2014a; Alejandra et al., 2018), coal gasification wastewater (Hou et al., 2016; Xu et al., 2019), and oil-containing wastewater (Yang et al., 2012; Merma et al., 2020), among others. The three-dimensional electrode method is a type of electrochemical oxidation technology characterized by high mass transfer efficiency, large specific surface area, good electrical conductivity, and low environmental pollution. Therefore, the three-dimensional electrodes have received increasing attention in the treatment of wastewater difficult to treat by conventional methods. On the other hand, the photocatalytic oxidation technology is considered an ideal and reliable contamination control method, to this end, nano-TiO_2_ is a photocatalyst widely investigated in photocatalysis (Parussulo et al., 2009; Park et al., 2020). However, its applications have been limited due to multiple factors, including strong dispersion of the light source, weak light intensity, elevated recombination probability of photo-generated electrons and holes, and high concentration of contaminants (Jay et al., 2019; Camacho-Muoz et al., 2020). To address these challenges and enhance the benefits of each technology, nano-TiO_2_ catalysts have been combined with the electrochemical oxidation technology to design appropriate three-dimensional electrodes for combining photocatalysis with electrocatalysis (Song et al., 2019; Arab et al., 2020). This process would not only inhibit the recombination of photo-generated electrons and holes but also greatly enhance the current efficiency, space-time yield, and electrolysis efficiency of the three-dimensional electrode system. Nevertheless, constructing efficient and stable three-dimensional photoelectric systems is still challenging. Moreover, owing to the good performance of the gasification slag, it has a certain electrical conductivity in the preparation of the particle electrode as raw material in the treatment of the catalytic technology, which could solve the problem of high cost of catalyst and complicated preparation processes (Wang et al., 2014b; Yan et al., 2014).

After considering the advantages and disadvantages of both photocatalytic and electrochemical oxidation technologies combined in three-dimensional electrodes, a Ti/RuO_2_-IrO_2_-SnO_2_-CeO_2_ anode was constructed on a pure Ti sheet by the anodizing method. An external ultraviolet (UV) lamp was used as a light source for the designed photoelectrical degradation system. The effects of multiple factors were evaluated by degrading a model organic contaminant MB in solution to mitigate the limitations of combined photocatalytic with electrochemical technology in terms of the difficult separation of the catalyst, the facile recombination of electrons and holes, the high energy consumption, and low current efficiency of electrochemical oxidation technology. Furthermore, numerous experiments were conducted on resource utilization since coal gasification slag contains unburned residual carbon and metal oxide minerals under high temperatures (Guo et al., 2020a; Guo et al., 2020b; Miao et al., 2020; Guo et al., 2022). Overall, novel insights into the design of three-dimensional photoelectrical systems for highly efficient catalytic degradation of organic contaminants were provided, promising for wastewater treatment in both theory and practice.

## 2 Experimental

### 2.1 Materials

Raw materials like anhydrous ethanol, Butyl titanate, Ferric nitrate, Hydrochloric acid, Sodium hydroxide, Methylene blue, Sodium chloride, Potassium chloride, Sodium sulfate, Sodium carbonate, Isopropanol, *n*-Butanol, Cerium nitrate, Ruthenium trichloride, Tin tetrachloride, Chloroiridic acid were purchased from Tianjin Beilian Chemical Reagents Company in analytical grade, and used as received without further purification. The coal gasification slag (CGS) was obtained from the Yanzhou Coal Industry Yulin Energy and Chemical Company. Titanium sheets (dimensions 50 mm × 30 mm × 2 mm) were purchased from Shenzhen Quanfu Metal Material Co., Ltd., China. These sheets were utilized as cathodes for the preparation of Ti/RuO_2_-IrO_2_-SnO_2_-CeO_2_ anode.

### 2.2 Methods

#### 2.2.1 Preparation of Ti/RuO_2_-IrO_2_-SnO_2_-CeO_2_ electrode

The preparation process consisted of first dissolving cerium nitrate (Ce(NO_3_)_3_) and tin tetrachloride (SnC1_4_) in isopropanol to form a solution with a concentration of 0.5 mol·L^−1^. Afterward, equal amounts of isopropanol and *n*-butanol were mixed, and a few drops were used as a solvent for the ruthenium and iridium salts. In other words, this mixture was used to prepare the solutions of ruthenium trichloride (RuC1_3_) and chloroiridic acid (H_2_IrC1_6_) at the concentration of 0.5 mol·L^−1^. According to the previous ratio, four samples of solution bottles were mixed to obtain the coating solution for the anode. Next, the pre-treated titanium sheet was coated with the prepared anodic coating solution followed by drying at 120°C for 10 min and then sintering at 450°C for 10 min. The coated sheet was then removed from the oven and cooled down to room temperature. The coating procedure was repeated until the number of coatings reached a pre-determined value. After the last coating, the sheet was dried for 10 min and sintered at 470°C for 2 min before cooling down to room temperature, thereby concluding the preparation of the dimensionally-stable anode (DSA).

#### 2.2.2 Preparation of Fe^3+^-TiO_2_@CGS particles

The basic characteristics of coal gasification slag (CGS) were conducted according to Chinese National Standard GB/T 212-2008, and the results are presented in Table 1. The preparation process consisted of first weighing a certain amount of CGS followed by drying at 150°C for 3 h. A certain amount of sodium carbonate was then added and sintered in a tubular furnace. Afterward, the mixture was heated to 800°C for 2 h under nitrogen for activity. To obtain the pretreated coal gasification slag after activation, the coal gasification residues were subjected to acid leaching and alkali leaching in a solution containing 4 mol/L hydrochloric acid and 2 mol/L sodium solution.

**TABLE 1:** Basic characteristics of coal gasification slag.

Sample	Proximate analysis (wt%)				Ultimate analysis (wt.%daf)				
	M_ad_	A_ad_	V_ad_	FC_ad_	C	H	N	S	O^*^
CGS	3.04	44.92	6.98	46.64	85.77	1.63	0.32	2.11	10.19

Ar, as-received basis; d, dry basis; daf, dry ash-free basis;* by difference.

For reagent A, anhydrous ethanol (18 mL) and Fe(NO_3_)_3_·9H_2_O (5.0 mmol, 2.03 g) were mixed under magnetic stirring. The reagent B was then prepared by adding tetrabutyl titanate (52.8 mmol, 18 mL) to anhydrous ethanol (36 mL) under magnetic stirring. Finally, both reagents A and B were mixed and stirred for 30 min to yield TiO_2_ sol, which then was mixed with 10 g of coal gasification slag, stirred for 1 h, and allowed to stand for 24 h. The filtered product was dried in a drying oven at 100°C followed by calcination in a tube furnace at 500°C under a nitrogen atmosphere. The resulting cooled product was pulverized in a mortar to yield Fe^3+^-TiO_2_@CGS particles prepared by the sol-gel method.

#### 2.2.3 Photoelectric degradation properties of MB

**FIGURE 1:**
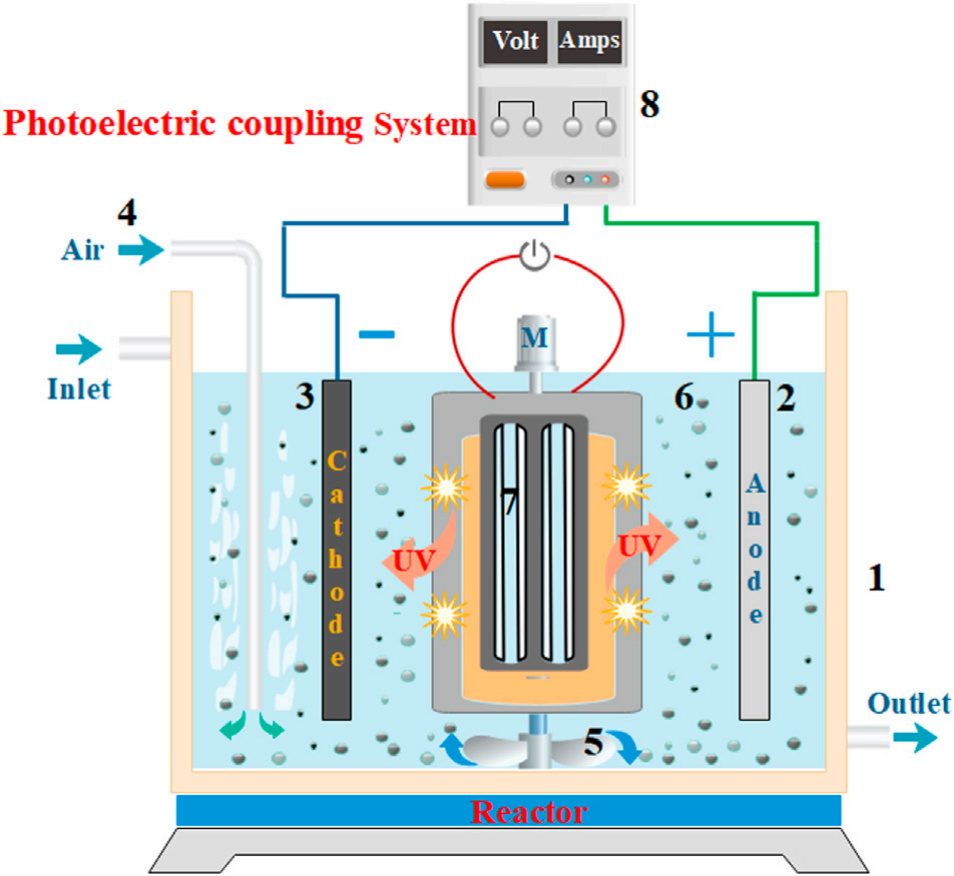
A diagram showing the wastewater treatment instrument of the photoelectrical system with a three-dimensional electrode. (1) Water tank, (2) DSA anode, (3) Titanium cathode, (4) Gas tube, (5) Mixer, (6) Fe^3+^-TiO_2_@CGS particles, (7) Light source, and (8) DC power supply.

The photoelectric degradation of MB in an aqueous solution was carried out in a photoelectrical degradation reactor under darkroom conditions (Figure 1). In a typical process, 500 mL of aqueous MB solution at a concentration of 600 mg·L^−1^ was placed in a beaker equipped with a magnetic stirrer. Different amounts of the supporting electrolytes (NaCl, Na_2_CO_3_, and Na_2_SO_4_) were added to the reactor for adjusting the conductivity of the solutions to 6,000 μS·cm^−1^ (3–5 g electrolyte solid is first added until the conductivity approach to 6,000 μS·cm^−1^, and then the saturated solution of the electrolyte is used for fine tuning). The pH of each solution was adjusted to the required value (3, 5, 7, 9, and 11) by adding either 4 mol·L^−1^ hydrochloric acid or 2 mol·L^−1^ aqueous NaOH solution. An amount of Fe^3+^-TiO_2_@CGS (5.0 g) particles was then added to the reaction flask of the photoelectrical reactor before placing it in the dark box of the reactor. A voltage-stabilized power supply and a 600 W UV lamp as the light source were next turned on for the reactions to take place before taking out for testing at certain time intervals. In the degradation experiment, 5 mL suspension was extracted from the reactor in a certain time, and then we used the UV-vis absorption spectra to analysis the existence of the MB. In addition, according to the Beer’s law: degradation rate (%) = (*C*_*0*_-*C*_*t*_)/*C*_*0*_ × 100% was calculated, where *C*_*0*_ is the initial absorbance of MB solution, *C*_*t*_ is the instantaneous absorbance of MB solution after a period of time. The degradation rate of MB was calculated from the absorbance measured by spectrophotometry.

### 2.3 Instrumentation

The Zeiss Sigma 300 scanning electron microscope (SEM, Zeiss, Germany) and transmission electron microscope (JEM, 2100F TEM (JEOL, Japan) were used for morphology and elemental composition analyses of the Ti/RuO_2_-IrO_2_-SnO_2_-CeO_2_ electrode and Fe^3+^-TiO_2_@CGS particles. The concentrations of MB in the solution samples were analyzed by spectrometry (UV-2450, Shimadzu, Japan). The absorption/desorption curves and pore size distributions of Fe^3+^-TiO_2_@CGS three-dimensional electrodes were analyzed by BET specific surface area (CN61M/SZB-9, Replete, China). The functional groups on the samples were tested by fourier transform infrared spectrometer (FT-IR, IS50, Nicolet, America). X-ray diffraction (XRD measurements were recorded by a Rikagu diffractometer (D_8_ Advance, BRUKE, Germany) with Cu-radiation (*λ* = 1.54178 Å) at 40 kV voltage and 30 mA current. The X-ray photoelectron spectroscopy (XPS) profiles were obtained on a Scientific ESCALAB 250Xi photoelectron spectrometer (Thermo Fisher, America).

## 3 Results and discussion

### 3.1 Morphology and composition of as-prepared Ti/RuO_2_-IrO_2_-SnO_2_-CeO_2_ electrodes

**FIGURE 2:**
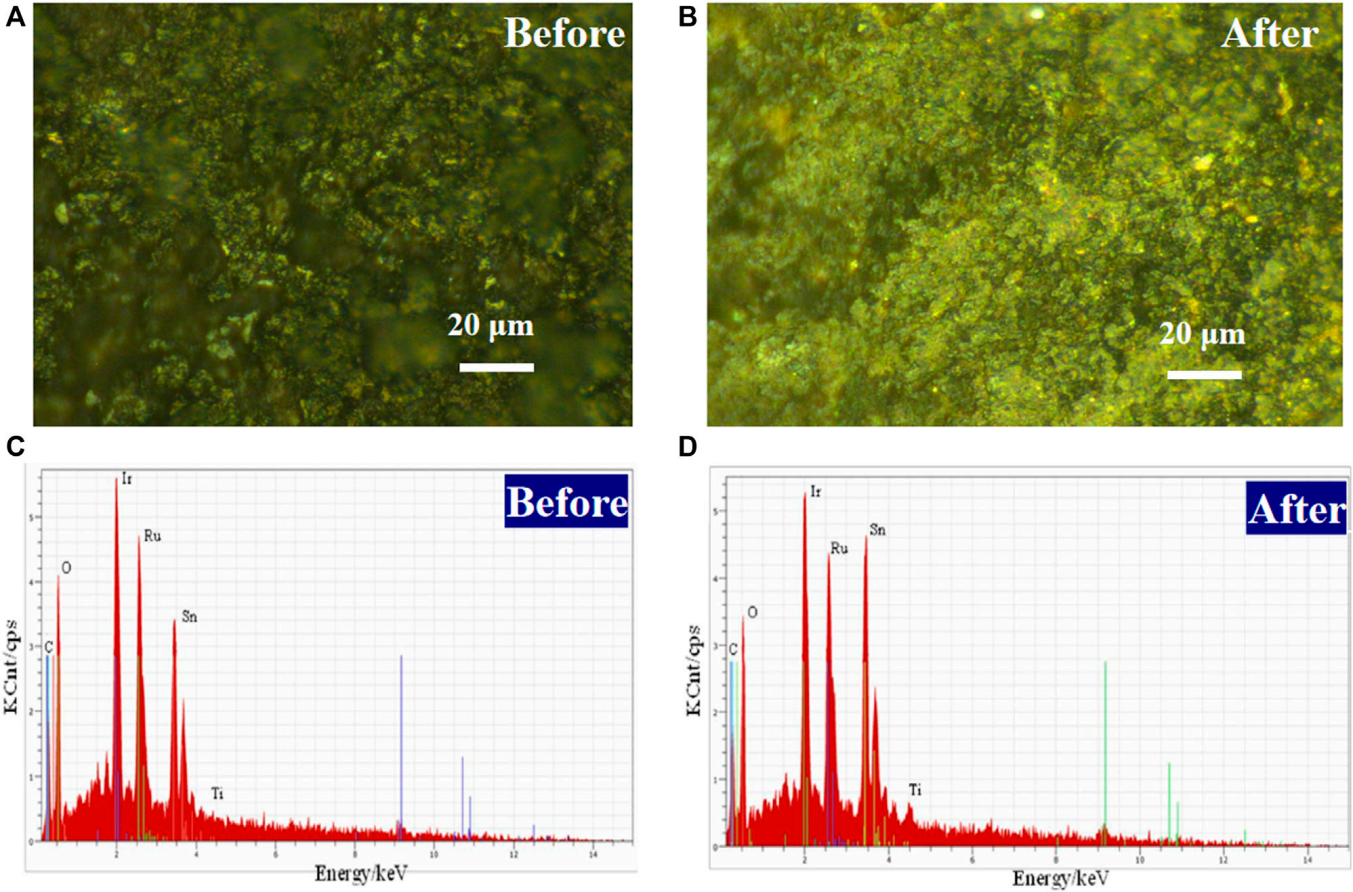
SEM micrographs and EDS spectra of DSA before **(A,C)** and after **(B,D)** used in the system.

The microstructures of the Ti/RuO_2_-IrO_2_-SnO_2_-CeO_2_ electrodes were viewed by SEM and the results are shown in Figure 2A. A fragmented structure containing cracks and a dense structure without cracks were observed on the electrode surface. These features were caused by the difference in thermal expansion coefficients of the metal coating and metallic titanium substrate. The formation of the cracked fragmented structure may be beneficial in terms of increasing the specific surface area of the electrode, facilitating contact with contaminants, and improving electrocatalytic performance. More importantly, the presence of the dense structure could enhance the gas erosion resistance of the electrode surface, which would effectively alleviate the penetration of O_2_ and electrolyte into the coating and titanium substrate, thereby inhibiting anode failure and extending its lifetime. The SEM micrographs of the electrode after use revealed no obvious changes, indicating the good stability of the prepared DSA (Figure 2B).

The elemental composition of the DSA coating surface was determined by EDS (Figures 2C,D). The surface of the coated electrode was mainly composed of five elements (Sn, Ru, O, C, and Ir), while the element Ce was absent in the spectra. This can be attributed to the incomplete dissolution of Ce in isopropanol, resulting in lower Ce content in the coating. According to literature reports (Lassali et al., 2000), the addition of iridium oxide into the coating may effectively reduce the chlorine evolution potential of the electrode, as well as enhance the corrosion resistance of the coating toward the oxygen evolution reaction, thereby enhancing the lifetime of the electrode. The addition of Sn-containing compounds could improve the formation of multi-crystals grain on the coating surface, thereby raising the specific surface area. Moreover, Ru and Ce, as active elements in the coating, can enhance the electrocatalytic activity of the anode.

### 3.2 Properties of Fe^3+^-TiO_2_@CGS three-dimensional electrodes

**FIGURE 3:**
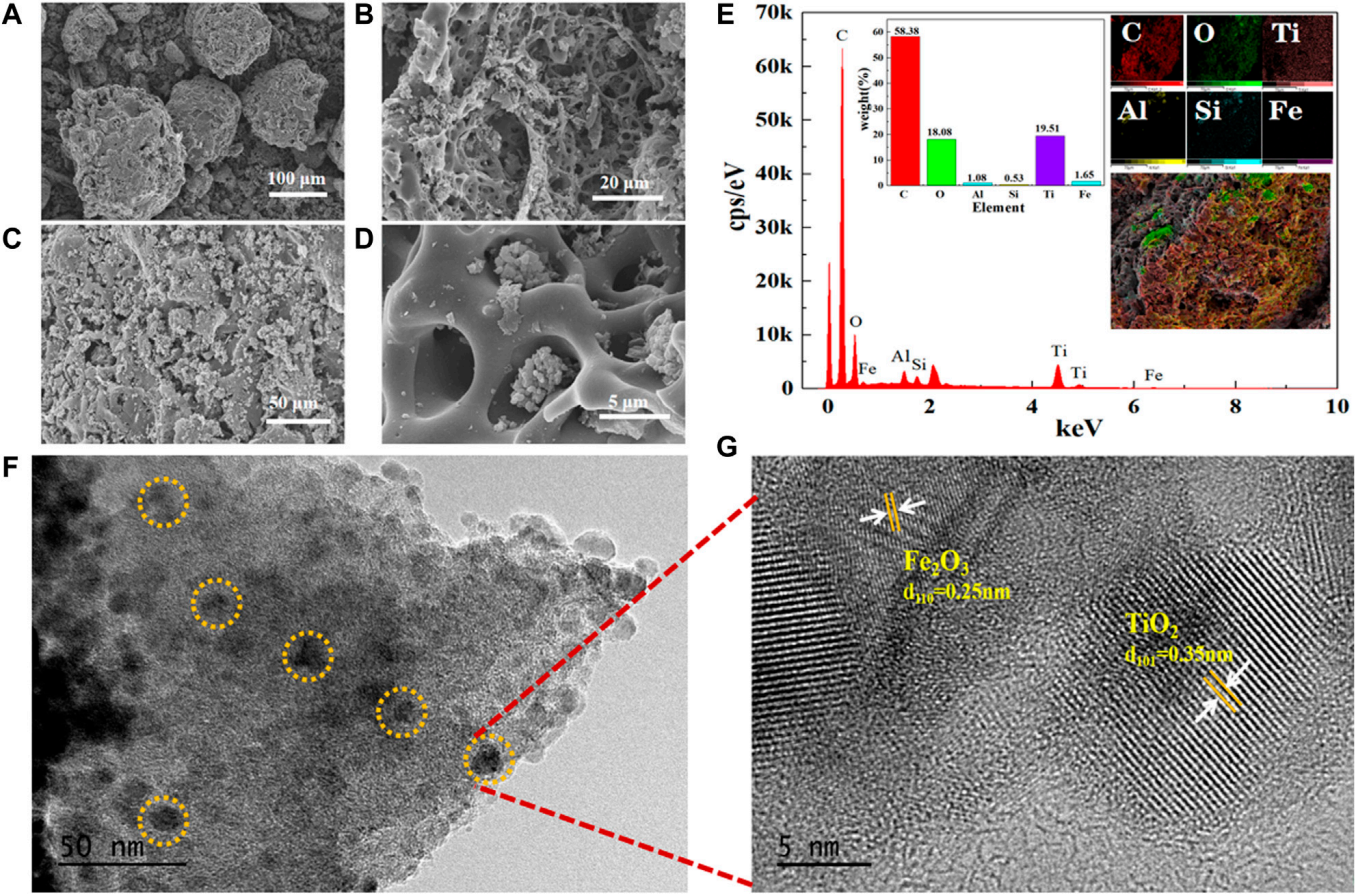
**(A–D)** SEM images, **(E)** EDS spectra, and **(F–G)** TEM images of Fe^3+^-TiO_2_@CGS particles.

The SEM micrographs of Fe^3+^-TiO_2_@CGS particles prepared by the sol-gel method revealed carbon in CGS existing in a flocculent amorphous morphology (Figures 3A–D **)**, with a relatively well-developed porous structure on the surface. In Figure 3C, white crystals looked uniformly distributed on CGS particles without agglomeration. The average particle size was estimated to 10 nm, and the structure of the particles appeared denser, indicating Fe^3+^-doped TiO_2_ widely distributed on the CGS carrier surface. Some TiO_2_ particles were embedded in the micropores of CGS (Figure 3D **)**, showing the pore size of CGS compatible with the size of TiO_2_ crystals. However, the adsorption channels inside CGS were not completely blocked. Also, the dimensions of some TiO_2_ grains looked larger than the surface micropores of CGS, hindering them from entering the micropores. Therefore, the as-prepared Fe^3+^-TiO_2_@CGS particles may exhibit a certain adsorption capacity during “absorption + photocatalysis + electrocatalysis” process of Fe^3+^-TiO_2_@CGS particles in the photoelectrical system designed for wastewater degradation.

As shown in Figure 3E, the gasification slag contained C, O, Ti, Si, Al, and Fe elements, in addition, the weight percentage of C and O elements were estimated to 58.38% and 18.08%, respectively. Thus, the prepared particles were successfully loaded on the Fe^3+^-doped TiO_2_. Moreover, the TiO_2_ particles were uniformly distributed on the CGS surface (TEM micrographs of Fe^3+^-TiO_2_@CGS particles, Figures 3F,G **)**. The lattice spacings of 0.35 nm and 0.25 nm were also kept well with the facet of TiO_2_ (101) and Fe_2_O_3_ (110), respectively (Figure 3G). Therefore, TiO_2_ and Fe_2_O_3_ nanoparticles were successfully loaded at the pores of CGS particles, confirming the feasibility of the preparation of Fe^3+^ -TiO_2_ @ CGS particles.

**FIGURE 4:**
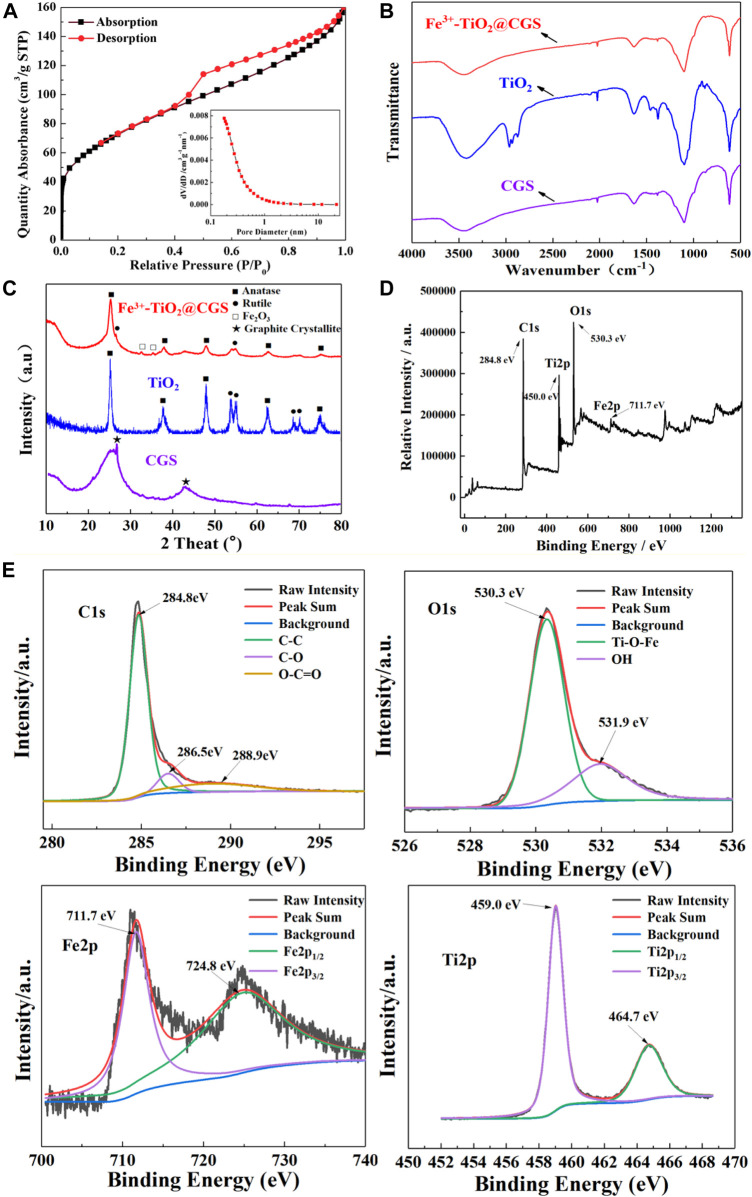
**(A)** Absorption and desorption curves of Fe^3+^-TiO_2_@CGS particles (the inset shows the pore size distribution curve); **(B)** FT-IR of Fe^3+^-TiO_2_@CGS three-dimensional electrode; **(C,D)** XRD and XPS full spectra of Fe^3+^-TiO_2_@CGS three-dimensional electrode; **(E)** XPS spectrum (C1s, O1s, Fe2p and Ti2p) of Fe^3+^-TiO_2_@CGS three-dimensional electrode.

The absorption-desorption curves and pore size distribution profiles of Fe^3+^-TiO_2_@CGS particles are presented in Figure 4A. The specific surface area, the average pore diameter, and the pore volume of Fe^3+^-TiO_2_@CGS particles were determined as 264.3259 m^2^·g^−1^, 0.247755gnn, and 0.247138 cm^3^·g^−1^, respectively. Hence, the pores remained incompletely occupied after doping the gasification slag with Fe^3+^-loaded TiO_2_. Table 2 revealed a decline of 59.01% in specific surface area of Fe^3+^-TiO_2_@CGS particles after loading. Also, all gasification slag before and after loading displayed flourishing pore structures. Therefore, the high microporous structure of gasification slag was not destroyed during the doping process, conducive to the full contact between particle surface and pollutants.

**TABLE 2:** Specific surface area and pore structure parameters of gasification slag.

Sample	Specific surface area (m^2^·g^−1^)	Pore volume (cm^3^·g^−1^)	Pore size (gnm)
CGS	644.3206	0.612210	4.33092
Fe^3+^-TiO_2_@CGS	264.3259	0.247138	0.247755

To further confirm the structures of CGS and Fe^3+^-TiO_2_@CGS, the FT-IR spectra were obtained. In the FT-IR sepctra (Figure 4B), it is found that the main skeleton of CGS is carbon-based material with corresponding vibration peaks. After the formation of Fe^3+^-TiO_2_@ CGS composite material, the structure changes slightly with some vibration peaks disappear or appear. Combined with TEM and XRD experimental results, it reveals that the composite material is prepared successfully.

As shown in Figure 4C, it can be seen that there are two obvious characteristic peaks at 25.8° and 44.7°, assigned to the diffraction peaks of graphite crystallites of the gasification slag. When TiO_2_ is loaded, the diffraction peaks are revealed in the composite material with the diffraction peak of Fe_2_O_3_, the reason is that the main skeleton of Fe^3+^-TiO_2_@CGS composite material is tends to be amorphous structure compared with the raw materials. This phenomenon indicates that TiO_2_ and Fe^3+^ particles are successfully composited on the CGS along with good stability and modifiability. Furthermore, the XRD patterns of the as-prepared Fe^3+^-TiO_2_@CGS particles revealed peaks at 2*θ* of 25.24°, 37.94°, 48.03°, 54.97°, 62.58°, 69.96°, and 74.98° (Figure 4C). These peaks can be attributed to anatase crystal planes (101), (004), (200), (105), (204), (220), and (215), consistent with the characteristic peaks of TiO_2_ standard card (JCPDS 021–1272). The peaks at 2*θ* of 26.51° and 35.32° were assigned to the rutile crystal plane (110) and (101), respectively. Note that characteristic peaks corresponding to the TiO_2_ standard card (JCPDS 021–1276) were consistent with experimental results. The peaks at 2*θ* of 32.48° and 42.75° were linked to the crystal planes (104) and (113), respectively. The characteristic peaks corresponding to the Fe_2_O_3_ standard card (JCPDS 001–1053) were also consistent with experimental data, indicating gasification slag successfully loaded on nano-TiO_2_ and mainly composed of anatase crystals. Fe_2_O_3_ crystals may exist, and Fe^3+^ was not fully infiltrated into TiO_2_ as no obvious characteristic peak of amorphous carbon was observed. The latter may be due to the weak peak intensity of amorphous carbon, which was overlayed by TiO_2_ characteristic peaks. The XPS diagram of Fe^3+^-TiO_2_@CGS showed four characteristic peaks of C, O, Fe and Ti (Figure 4D). The peaks of C1s, O1s, Fe2p, Ti2p located at 284.8 eV, 530.3 eV, 711.7 eV and 459.0 eV. In Figure 4E, the peak at 284.8 eV is corresponded to the C1s, and the others at 286.5 eV, 288.9 eV are assigned to the C-O and O-C═O bonds of CGS. The peak at 530.3 eV of O1s, which indicated the forming of Ti-O-Fe bond in the TiO_2_ lattices. The presence of -OH group was ascribed to the peak at 531.9 eV. Furthermore, the peaks at 711.7 eV and 724.8 eV in Fe2p corresponded to Fe2p_3/2_ and Fe2p_1/2_ of the metal-ion Fe^3+^. There are two peaks at 459.0 eV and 464.7 eV in the spectrum of Ti2p, which corresponding to Ti2p_3/2_ and Ti2p_1/2_ (Hassan et al., 2016; Singh et al., 2019). In Figure 4D, Fe^3+^ also entered the lattice of TiO_2_, and the introduction of Fe^3+^ played an irreversible role in the electron trap. In other words, defects were introduced into the lattice after replacement of Ti^4+^ by Fe^3+^. The cross between Fe2p state and TiO_2_ crystal valence band broadened the valence band and narrowed the energy gap, which can reduce electron-hole recombination and effectively improve photocatalytic efficiency.

### 3.3 Degradation rate of MB under different conditions

To assess the photoelectric coupling effect of the three-dimensional electrode, the degradation of MB at a concentration of 600 mg·L^−1^ and volume of 500 mL was carried out in the photoelectrical reactor (Figure 1). The process was performed either with photocatalysis under UV light, electrocatalysis with the three-dimensional electrode, or a combination of both in synergistic photoelectric catalysis experiments. The conditions consisted of a Fe^3+^-TiO_2_@CGS particle of 5 g, NaCl electrolyte concentration of 0.15 mg·L^−1^, treatment time of 1 h, and current density of 15.63 mA·cm^−2^.

**FIGURE 5:**
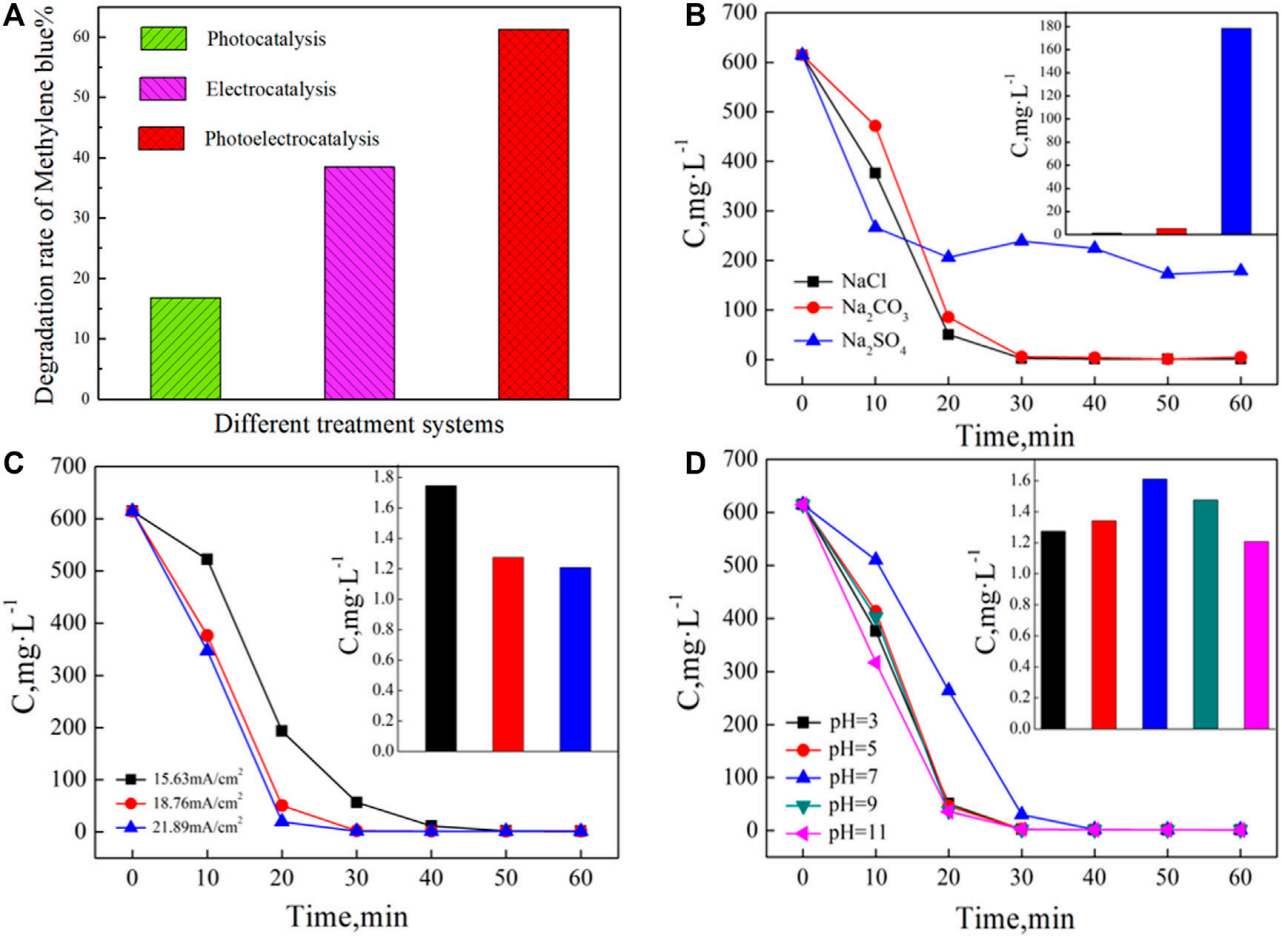
**(A)** The degradation rate of MB in different treatment systems,**(B–D)** Effects of the supporting electrolyte, current density,and initial pH on the degradation rates of MB, respectively.

From Figure 5A, the decomposed MB fractions were estimated to 16.8% under UV irradiation alone and 38.5% when employing electrocatalysis alone. The decomposition of MB using the photoelectric synergistic catalysis system *via* a three-dimensional electrode was recorded as 61.3%, a value higher than the sum of both separately conducted catalytic methods. The synergistic factor (C = η_3_/(η_1_+η_2_)×100%) was calculated as 1.11, indicating an obvious photoelectric synergistic effect in this system (Xu et al., 2008; Hou et al., 2017a).

The conditions of the (Gan et al., 2008) photoelectrocatalytic would influence the treatment effect of wastewater, including electrolyte type, current density, and initial pH of the solution. Based on previous works (Zeng et al., 2022; Zhang et al., 2022), an in-depth analysis of the supporting electrolyte, current density, and initial pH of the solution was carried out. The influencing mechanism of the degradation effect of methylene blue, as well as the optimal operating conditions, were obtained.

In order to study the effects on the degradation of MB, sodium salts (NaCl, Na_2_CO_3_, and Na_2_SO_4_) were employed as supporting electrolytes in separate experiments. For each experimental condition, the initial MB concentration was kept at 600 mg·L^−1^ along with 5 g of Fe^3+^-TiO_2_@CGS particles and a high-pressure Mercury lamp at a power of 600 W. The system was also purged with at the flow of 200 L·h^−1^. At the initial pH 3, the electrolytes were added to adjust the conductivity to 6,000 μS·cm^−1^, and the reaction was carried out for 60 min. Figures 5B–D gathers the results of the effects of process conditions, including electrolyte type, current density, and initial pH of the solution on the degradation rates of MB. The plots of MB concentration as a function of time in the photoelectric catalytic system containing different supporting electrolytes are provided in Figure 5B. After 10 min reaction, the concentration of MB decreased significantly in the presence of all three supporting electrolytes. After the addition of Na_2_SO_4_ to the system, the decomposition rate was accelerated. However, the degradation in NaCl and Na_2_CO_3_ as supporting electrolytes looked more effective than in Na_2_SO_4_ for the reaction proceeding for 20 min. The calculated degradation yields at 30 min reaction time were 99.78%, 99.19%, and 61.22%, with insignificant performance differences between NaCl and Na_2_CO_3_. The introduction of Cl^−^ in alkaline Na_2_CO_3_ solution as an electrolyte might be explained by the increase in the pH value to 10. When 4 mol·L^−1^ hydrochloric acid was added to the solution, the pH value returned to 3. Therefore, the introduced Cl^−^ anions might determine the decomposition performance of the system. In the following 30 min reaction, the degradation in the three supporting electrolytes ceased, with final degradation yields determined as 99.95%, 99.32%, and 71.08%, respectively.

The conductivity of the solution may significantly influence the degradation process of organic matter (Solarska et al., 2006). The addition of the supporting electrolyte in the coupled photoelectric process could enhance the current efficiency, resulting in the effective degradation of wastewater pollutants. In the designed photoelectrical treatment system for MB, the reactions of Cl^−^ can be promoted under photoelectric coupling by introducing Cl^−^ to the supporting electrolyte. Certain concentrations of active chlorine may induce strong oxidizing properties (Wang et al., 2007). Also, large numbers of active substances, such as HO·, O^−^, and Cl^−^ may be generated under photocatalysis, conducive to increasing the degradation rates of organic matter (reactions 1–4).

$$2{Cl}^{-}-2e^{-}\to {Cl}_{2}$$ (1)

$${Cl}_{2}+H_{2}O\to HClO+HCl$$ (2)

$${Cl}_{2}+HR\to RCl+HCl$$ (3)

$$HClO+HR\to RCl+H_{2}O$$ (4)

Using Na_2_SO_4_ as the supporting electrolyte, MB was decomposed at a certain degradation rate during the first 20 min of the reaction, leading to a decrease in the subsequent period. The reason for this might have to do with the generation of a certain amount of H_2_O_2_ from SO_4_^2-^ in the Na_2_SO_4_ system *via* reactions (5) and (6) under photoelectric excitation. In turn, the formed H_2_O_2_ may promote the degradation of MB solution. At prolonged reaction times, H_2_O_2_ would gradually decompose in the presence of light, and SO_4_^2-^ can capture H^+^ or HO· produced in the system to generate relatively weak oxidizing agent SO_4_^2-^ *via* reactions (7) and (8) (Li et al., 2020). These mechanisms would explain the degradation rate in solution through this system, indicating the inorganic salt of NaCl was a better supporting electrolyte in the photoelectrical treatment of MB than Na_2_CO_3_ and Na_2_SO_4_.

$${SO}_{4}^{\;2-}-2e^{-}+hv\to S_{2}O_{8}^{\;2-}$$ (5)

$$S_{2}O_{8}^{\;2-}+2H_{2}O\to 2{HSO}^{4^{-}}+H_{2}O_{2}$$ (6)

$$HO\cdot +{SO}_{4}^{\;2-}\to {SO}^{4^{-}}+{OH}^{-}$$ (7)

$$h^{+}+{SO}_{4}^{\;2-}\to {SO}^{4^{-}}$$ (8)

To clarify the effect of the current density on the degradation of MB, the same above reaction conditions were employed to study the influence of the supporting electrolyte by selecting NaCl as the electrolyte. The reactions were carried out for 60 min at current densities of 15.63, 18.76, and 21.89 mA·cm^−2^.

As shown in Figure 5C, the current density significantly affected the photoelectrical degradation of MB in solution. A good performance was achieved for both degradation efficiency and reaction rate. After 30 min reaction time, the degradation yield at the current density of 18.76 mA·cm^−2^ exceeded 99%, while the same yield was achieved after 50 min at a current density of 15.63 mA·cm^−2^. Therefore, within a certain concentration range, MB can be effectively degraded at increased current densities. However, the degradation effect varied slightly when the current density further rose from 18.76 mA·cm^−2^–21.89 mA·cm^−2^.

The observed variation can be explained by the increase in the amount of electricity passing through the photoelectrical system in the same period with the current density, promoting the generation of active chlorine from Cl^−^. This considerably contributed to the degradation of wastewater, leading to better degradation of organic matter. Furthermore, the increase in current density provided sufficient energy for the reactions (9) and (10) to enhance the amount of HO· in the system, thereby promoting the degradation of organic matter. However, the excess H_2_O_2_ consumed strongly-oxidizing reagents, such as photo-generated holes or HO· in the system, inhibiting the degradation of wastewater. Consequently, the reaction rate gradually decreased or even ceased to occur. However, higher current densities in the coupled photoelectric process under laboratory conditions shortened the reaction time, enhancing the difficulty of reaction occurrence and appearance of intermediate products. Besides, the voltage increase with current density would more likely damage the electrode surface and greatly shortens its lifetime. By considering the above factors, the coupled photoelectric degradation of MB solution would be optimal to carry out at a current density of 18.76 mA·cm^−2^, which was used for subsequent experiments.

$$O_{2}+2H^{+}+2e^{-}\to H_{2}O_{2}$$ (9)

$$H_{2}O_{2}+hv\to 2HO\cdot$$ (10)

To investigate the effects of different initial pH values on the degradation of MB solution, the reaction was carried out at initial pH values of 3, 5, 7, 9, and 11 for 60 min under the same above conditions using NaCl as supporting electrolyte at the current density of 18.76 mA·cm^−2^.

In the photoelectric catalysis process, Figure 5D shows the treatment effects of different initial pH values on MB decomposition. The reaction process of MB decreased significantly under all five conditions. However, the degradation rate first declined and then rose with pH value. In the neutral solution, the degradation displayed relatively low rates with a yield of only 61.38% after a reaction time of 20 min, while 90% decomposition was achieved under other conditions in the same time frame.

For the photoelectrical degradation of MB solution, the initial pH of the solution would affect the degradation of organic matter, as well as the surface charge of the catalyst to a certain extent. At pH < 7, H^+^ concentration existed at a relatively high levels, thereby reaction (9) would naturally proceed in the forward direction, leading to increased H_2_O_2_ concentration in the solution. The generated H_2_O_2_ underwent reaction (10) to form large amounts of HO· under the photoelectric process, thereby enhancing the ability of the degradation of MB in solution. As pH rose, OH^−^ under alkaline conditions promoted reaction (11) with dissolved oxygen generated under aeration conditions, thereby generating HO_2_· that can directly oxidize organic matter. HO_2_· also underwent reactions (12) and (10) to generate HO·, which can degrade organic matter. Meanwhile, the protonated TiO_2_ surface was positively charged at low pH values, conducive to the transfer of photo-generated electrons to the TiO_2_ surface. At much higher pH values, the surface became more negatively charged, thereby promoting the transfer of holes from the inside of particles to the surface. Therefore, the photoelectrical degradation of MB exhibited higher reaction rates under both lower and higher pH values than under neutral conditions.

$$O_{2}+2{OH}^{-}\to 2{HO}_{2}\cdot$$ (11)

$$2{HO}_{2}\cdot \to H_{2}O_{2}+O_{2}$$ (12)

Overall, MB can effectively be degraded in solution by the designed photoelectrical reactor. The trend of the MB concentration varied under different conditions owing to the effects of supporting electrolyte, current density, initial pH of the solution, and other factors. At pH 11, current density of 18.76 mA·cm^−2^, and NaCl as the supporting electrolyte, the degradation yield of wastewater reached 99.98% after 60 min of photoelectrical treatment, demonstrating an outstanding degradation effect. At 60 min under these conditions, the instantaneous concentration of CO_2_ detected by the miniature detector was just only 3,386 ppm, indicating that the amount of CO_2_ released was very small, and a large amount of MB was converted into organic intermediates in solution.

### 3.4 UV-visible spectroscopy of MB

**FIGURE 6:**
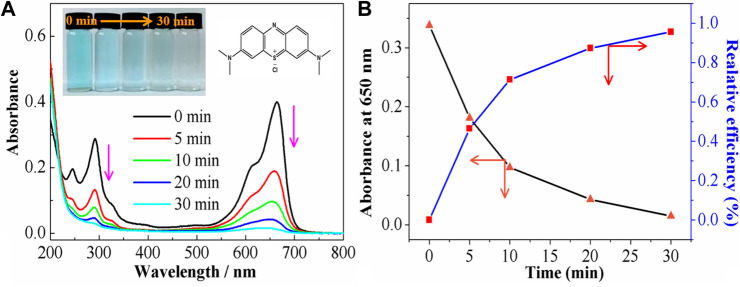
**(A)** Uv-vis spectra of the MB, and **(B)** the relative efficiency at the different degradation times.

During the initial 30 min of photoelectrochemical coupling treatment of MB solution in NaCl supporting electrolyte at pH 11 under a current density of 18.76 mA·cm^−2^, the color of the solution significantly decreased from light blue to colorless. To gain a better understanding of the changes in MB with reaction time, the solutions at different stages were diluted five times and then subjected to spectroscopy. As shown in Figure 6A, the initial solution of MB exhibited two obvious characteristic peaks at 664 nm and 292 nm, related to the chromophore structure (thiol) and aromatic ring structure absorption peak of MB, respectively (Xiang et al., 2017; Aritonang et al., 2018)^.^ The intensities of these peaks decreased with reaction time. In addition, MB was almost fully degraded after 30 min, consistent with the previous results. To further quantify the MB degradation capacity in a three-dimensional photoelectric system (Figure 6B), the MB degradation amounts were expressed by: R=(A_0_-A_t_/A_0_)×100%, where R is the MB degradation rate (Li et al., 2019; Wang et al., 2019), A_0_ represents the initial absorbance, and A_t_ is the absorbance at a certain time. Under optimal conditions, the degradation rate of MB reached 99.56% at 30 min. The three-dimensional photoelectric system showed large degradation capacities of MB dyes by absorption, electrochemical action, generated HO·, and molecular interactions. Such excellent performances testify to the usefulness of Fe^3+^-TiO_2_@CGS in the treatment of water pollution for target dye.

**FIGURE 7:**
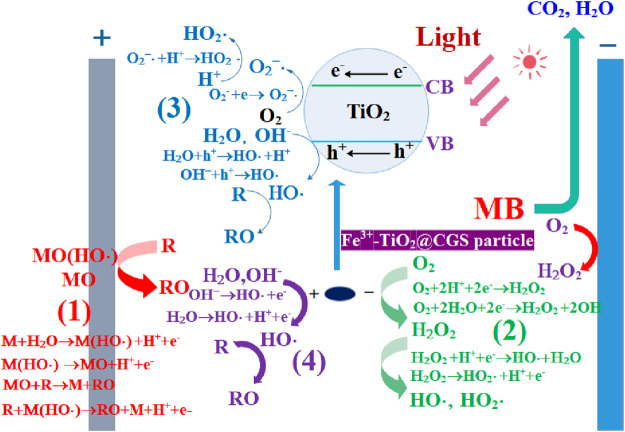
Mechanism diagram of MB degradation by three-dimensional electrode photoelectric system.

### 3.5 Mechanism of MB treatment by three-dimensional electrode photoelectric system

The degradation of MB was obvious in the constructed three-dimensional electrode photoelectric system. Based on relevant literature (Li et al., 2015; Qin et al., 2015; Hou et al., 2016; Hou et al., 2017b), as shown in Figure 7, the degradation effect may be related to several factors. The first had to do with direct anodic oxidation, in which the active metal component M on DSA anode surface can react with water to form free radicals, which when adsorbed on the electrode would form adsorbed free radicals M(HO·). The active metal component can also interact with free radicals to produce stronger oxides MO with higher valence. Finally, the organic functional group R of MB can directly be degraded on the anode surface through the oxidation of M(HO·) and MO attached to the anode surface. Second, in the three-dimensional electrochemical reaction system, oxygen would be generated by electrolysis and the dissolved oxygen would reduce at the anode to form the strong oxidizing substances H_2_O_2_, which, in turn, further decompose to form strong oxidizing free radicals HO· and HO_2_· to degrade MB. Third, TiO_2_ is an n-type semiconductor with band gap of 3.2 eV (anatase). When it receives ultraviolet light with a wavelength less than or equal to 387.5 nm, it will generate electrons and hole. The electron-hole h^+^ generated by the excitation of TiO_2_ in Fe^3+^-TiO_2_@CGS particles would react with the pollutants adsorbed on the catalyst surface to degrade MB. The H_2_O and OH^−^ adsorbed on the catalyst surface in solution would be in contact with the electron holes to form the active group HO·, conducive to degradation of MB. Meanwhile, O_2_^−^· and HO_2_· produced by the reaction of dissolved oxygen with photogenerated electrons could degrade MB. Fourth, Fe^3+^-TiO_2_@CGS particles in the three-dimensional electrode photoelectric system would be repolarized due to voltage excitation, similar to the anode of the system. The surface of the Fe^3+^-TiO_2_@CGS particles can adsorb MB and directly capture electrons of some unstable organic groups during the reaction, leading to the degradation of MB. In addition, HO· and HO_2_· produced in the reaction would degrade MB effectively. Therefore, the pore structure and adsorption capacity of Fe^3+^-TiO_2_@CGS particles were conducive to improving the degradation ability of MB.

The gasification slag with high carbon residue contents not only has an adjustable pores and functional groups that can enrich MB on its surface, but also can be used as a carrier for Fe^3+^ and TiO_2_ for the further preparation of Fe^3+^-TiO_2_@CGS particles. As is known to all, TiO_2_ is a semiconductor material in the system of photoelectric, electron transition from valence band to conduction band, it will produce strong reactive oxidation groups and holes, but also can further promote the ability of electrochemical reaction. In addition, the doping of Fe^3+^ contributes to the formation of Fe^3+^/Fe^2+^ in solution, which leads to the formation of Fenton reaction (Fe^2+^+H_2_O_2_→Fe^3+^+HO^−^ + HO·)at the cathode. And then the MB is oxidized to organic intermediates, and further degraded into CO_2_ and H_2_O under the action of photoelectric fields. Undoubtedly, the different components of Fe^3+^-TiO_2_@CGS particles are the fundamental factor for synergistic interaction of the absorption, photocatalysis, three-dimensional electrode electrocatalysis, and Fenton technology for the treatment of the industrial waste water. All in all, it will propose a basic idea for the treatment of the organic pollutants.

## 4 Conclusion

In summary, a novel three-dimensional photoelectric reactor system was successfully constructed and applied to study the effect of photocatalysis, electrocatalysis, and photoelectrical catalysis on the degradation of MB in solution. The cooperation of the photoelectric catalytic system with a three-dimensional electrode combined photocatalysis and electrocatalysis with a synergistic factor of 1.11. Furthermore, active substances (HO·, HO_2_·, and O_2_^−^·) were generated and acted as highly-oxidizing active chlorine species under electrochemical conditions of photocatalysis, greatly increasing the degradation rate of MB. As current density rose, the degradation efficiency of MB and the reaction rate showed remarkable enhancements. During the photoelectrical treatment of MB in solution, higher reaction rates were obtained under both lower and higher pH values when compared to neutral conditions. Overall, the proposed three-dimensional photoelectric reactor system looks promising for persistent organic pollutants.

## Data Availability

The original contributions presented in the study are included in the article/Supplementary Material, further inquiries can be directed to the corresponding authors.
